# Haemorrhoids and Anal Fissures in Pregnancy: Predictive Factors and Effective Treatments

**DOI:** 10.7759/cureus.53773

**Published:** 2024-02-07

**Authors:** Rebecca S Boughton, Caroline Brophy, Gillian Corbett, Sophie Murphy, Jacqui Clifford, Ann Hanly, Myra Fitzpatrick, Laoise O'Brien

**Affiliations:** 1 Obstetrics and Gynaecology, The National Maternity Hospital, Dublin, IRL

**Keywords:** obstetric, postpartum, pregnancy, anal fissure, haemorrhoids

## Abstract

Introduction

Haemorrhoids and anal fissures (HAF) are common in pregnancy and can severely affect the quality of life of those suffering from them. Despite the condition being common, there is limited evidence, formal guidelines or recommendations on treatment, and little is known about the natural course during pregnancy.

Methods

This was a prospective, observational cohort study conducted at a tertiary-referral university maternity hospital (The National Maternity Hospital, Dublin), conducted over a nine-month period. The first part of the study was a case-control study of antenatal patients over 34 weeks' gestation. The second part of the study involved a cohort of postnatal patients. Anonymous patient surveys were performed and analysed.

Results

Two hundred and fifty-eight patients were recruited into the study from the outpatient clinics and wards of one maternity hospital from April to December 2021. Of the antenatal patients, 82/184 (45%) of these patients had symptoms of HAF and 102/184 (55%) antenatal patients were unaffected, acting as controls. In addition, 74 affected postnatal patients were also included. In the affected antenatal group, 36/82 (44%) of patients had self-reported HAF (symptoms or signs of HAF); 50/82 (61%) of patients diagnosed with HAF on their own. 12/82 (15%) noticed symptoms in the first trimester, 25/82 (30%) in the second and 45/82 (55%) in the third. 142/184 (77%) of antenatal patients used conservative methods to manage their symptoms, including an increase in dietary fibre. 144/184 (78%) used medical treatments including suppositories. Only one patient had surgery. 70/156 (45%) of postnatal patients’ symptoms resolved within days, 42/156 (27%) in weeks and 44/156 (28%) within months.

Conclusion

HAF affect almost half of the pregnancies. Age over 35 was significantly associated with antenatal haemorrhoids or anal fissures. Concerningly, the majority of patients (64%) self-diagnose and manage the condition without either seeking or receiving guidance from medical professionals. In terms of the natural course of the disease, it was encouraging that 45% of patients’ symptoms resolved within a few days. This will help when counselling patients with distressing symptoms. Conservative measures such as increased dietary fibre, increased fluid intake and bath salts were effective in relieving symptoms for the majority of patients.

## Introduction

Haemorrhoids and anal fissures (HAF) are frequent in pregnant women and can reduce the quality of life of patients with them [1]. Up to 85% of pregnant women can have symptoms of HAF. Sixteen percent of patients can be affected in the long term, with symptoms six months after delivery [2,3]. In a prospective study of 280 pregnant women, just under half of the women had peri-anal disease during their pregnancy [4]. Despite the condition being common, there are limited formal guidelines, evidence or recommendations on treatment [5]. In addition, there is little known about the natural or predicted course of haemorrhoids and fissures during pregnancy. Women might not ask for medical advice due to not knowing the condition or because the condition is related to an intimate part of the body [1].

Haemorrhoids are variceal dilatations of the anal and perianal venous plexus [6]. There is anal cushion enlargement that can be symptomatic [7]. Haemorrhoids can be classified by prolapse: No prolapse (1st degree); prolapse on straining and spontaneous reduction (2nd degree); prolapse on straining and requirement for manual reduction (3rd degree); prolapsed and irreducible (4th degree) [8]. An anal fissure can be defined as a “split or tear in the anal canal” [5]. HAF occur more often in pregnancy due to pressure on the inferior vena cava with the increased abdominal cavity pressure, and also due to the increased circulating blood volume in pregnancy [7].

Whilst there have been guideline papers published on the management of haemorrhoids in general [9,10] and guidelines published on bleeding in pregnancy [11], there is a lack of evidence and guidelines specifically on the management of HAF in pregnancy. The aims of this study were as follows: to highlight the incidence of self-reported HAF during pregnancy in an Irish setting, to identify maternal and obstetric factors predictive of HAF in pregnancy, to describe the natural course of HAF symptomatology for patient counselling, and to describe the management options employed by Irish women, along with the patient-rated efficacy of these treatments.

## Materials and methods

This was a prospective observational cohort study conducted at a tertiary-referral university maternity hospital (The National Maternity Hospital, Dublin), conducted over a nine-month period (April to December 2021). The first part of the study was a case-control study of antenatal patients over 34 weeks’ gestation. The second part of the study involved a cohort of postnatal patients. Patients were recruited in both the outpatient and inpatient settings. Anonymous patient surveys were performed. Antenatal patients were approached in the general outpatient departments and the antenatal inpatient wards. Postnatal women were recruited consecutively from the postnatal wards but also from the dedicated postnatal clinics such as the POPPY perineal clinic where women with postnatal complications attend. A hard copy anonymous patient survey was used to collect the data from patients. Patients waiting for an outpatient appointment, or whilst on the ward, were approached, screened and counselled for recruitment. After time to consider, the researcher returned to the screened participants and obtained informed written consent.

Patients with haemorrhoids and/or anal fissures were compared against controls (those without these conditions). In the postnatal patients, only those with haemorrhoids and/or anal fissures were analysed, as they were a select cohort of postnatal women re-attending the hospital and the true incidence of postnatal haemorrhoids or fissures could not be derived. The patient survey was developed by the authors and approved by the ethics committee. Patient demographics were assessed including age, parity, number of vaginal deliveries and body mass index (BMI). Patient self-reported HAF information from prior to the current/recent pregnancy was collected, as well as, who diagnosed HAF. In the current/recent pregnancy, data were collected on how symptoms changed over time, as well as what treatments helped. Data on who diagnosed and managed the HAF symptoms were collected also.

Statistics and ethics

Data was compared between unaffected and affected women. Descriptive frequency analysis was performed on all outcomes. Categorical data was compared using the Chi-squared test to assess for statistically significant differences in the groups. Relative risk was assessed and 95% confidence intervals were also calculated (IBM SPSS Statistics for Windows, Version 28 (Released 2021; IBM Corp., Armonk, New York, United States)). Patient self-reported treatments were recorded and whether patients felt these treatments were effective or not. In postnatal patients, the natural progression of symptoms over time was recorded, as well as specifically what symptoms patients had. The study was approved by the Local Research Ethics Committee (EC38.2020).

## Results

Two-hundred and fifty-eight patients were recruited into the study from the outpatient clinics and wards of one maternity hospital from April to December 2021. Of the antenatal patients, 82/184 (45%) of these had symptoms of HAF and 102/184 (55%) antenatal patients were unaffected, acting as controls. In addition, 74 affected postnatal patients were also included in treatment efficacy analysis. In the affected antenatal group, 36/82 (44%) of patients had self-reported HAF (symptoms or signs of HAF). 50/82 (61%) of patients diagnosed with HAF on their own. 12/82 (15%) noticed symptoms in the first trimester, 25/82 (30%) in the second and 45/82 (55%) in the third. Table 1 displays the methods antenatal patients used to control their symptoms. It also highlights whether patients subjectively found these treatments to be useful or not. 142/184 (77%) patients used conservative methods to manage their symptoms, including an increase in dietary fibre. 144/184 (78%) used medical treatments including suppositories. Only one patient had surgery.

**Table 1:** Treatment strategies and their patient-reported efficacy

Strategy	Treatment Option	N Used	N Found Effective (Efficacy Rate)
Conservative	Baths – Sitz salt baths	22	20 (91.5%)
Conservative	Improved Dietary Fibre	58	51 (87.9%)
Conservative	Improved fluid intake	63	55 (87.3%)
Conservative	Ice packs	15	12 (80%)
Conservative	Manual Reduction	34	24 (70.5%)
Medical	Scheriproct suppositories	17	15 (88.2%)
Medical	Scheriproct topical	38	33 (86.8%)
Medical	Anusol	27	21 (77.8%)
Medical	Laxatives – Fybogel, Lactulose, Movicol	19	14 (73.7%)
Medical	Simple Analgesia	24	16 (66.7%)

**Figure 1:**
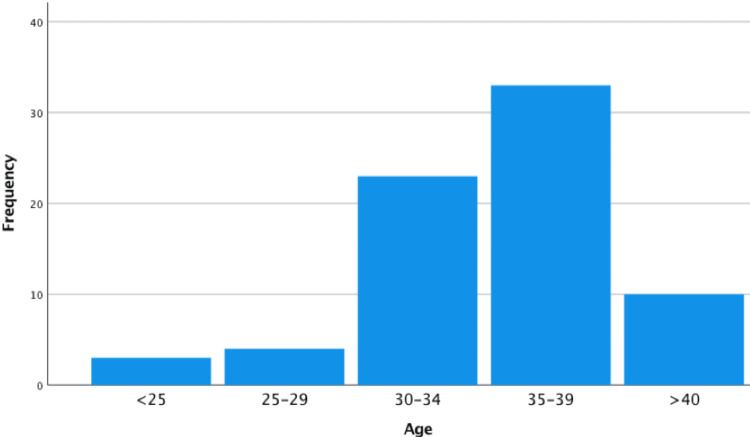
Age ranges of postnatal patients

**Figure 2:**
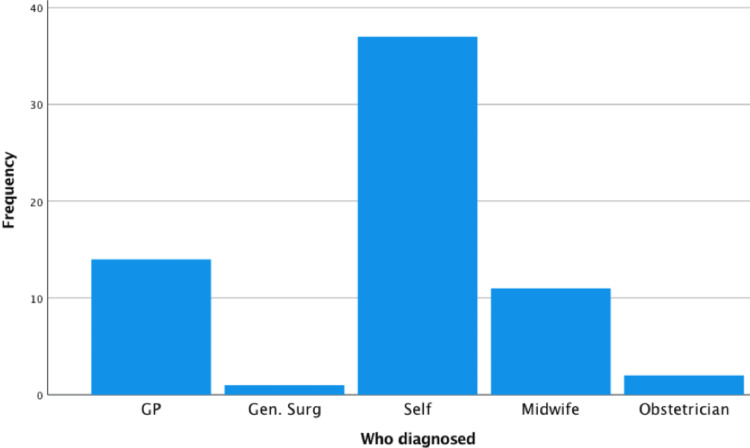
Diagnosis of the condition of haemorrhoids/anal fissures by different persons GP: General  practitioner

Demographics of antenatal patients with and without symptoms of HAF are displayed in Table 2. In patients with symptoms of HAF, there were significantly more patients older than 35 years, compared to the control group (69% (59/82) versus 26% (26/102), relative risk 3.0, 95% confidence interval 2.03-4.39). There were no other significant risk factors identified. In the postnatal group (with symptoms of HAF), the most common age group was 35-39 (70/156 (45%) patients see Figure 1). Body mass index (BMI) varied but most patients were of low BMI. 59/156 (38%) were of BMI <25, 47/156 (30%) were in the 25-29 range, 22/156 (14%) were in the 30-34 range, 6/156 (4%) 35-39 and one patient had a BMI of over 40. 29/156 (52%) of postnatal patients had haemorrhoids prior to the pregnancy or in a previous pregnancy and 11/156 (7%) had anal fissures before. In terms of diagnosis, 89/156 (57%) of patients diagnosed the condition themselves (see Figure 2). In most cases, this diagnosis was made by symptoms alone (103/156 (66%) of cases). Only 53/156 (34%) of patients had examination findings of haemorrhoids or anal fissures.

**Table 2:** Demographics/predictive factors for haemorrhoids or anal fissures during pregnancy The relative risks (RR) of the predictive factors are displayed, with the confidence intervals and p values. A p value of less than 0.05 was taken as significant and denoted with an asterix.

	Antenatal Affected (n=82)	Unaffected (n=102)	RR (95% Confidence Interval) * indicates p<0.05
Age > 35	59 (69%)	26 (26%)	2.8* (1.97-4.04), p<0.0001
BMI>25	50/71 (70%)	52/81 (64%)	1.1 (0.88-1.37), p=0.43
Parous	49 (56%)	51 (50%)	1.2 (0.93-1.57), p=0.19
Previous Vaginal Delivery	36 (44%)	37 (36%)	1.2 (0.86-1.76), p=0.29
Iron Supplementation	18 (22%)	20 (20%)	1.1 (0.64-1.97), p=0.69
Hypothyroidism	9 (11%)	7 (7%)	1.6 (0.62-4.11), p=0.28

**Figure 3:**
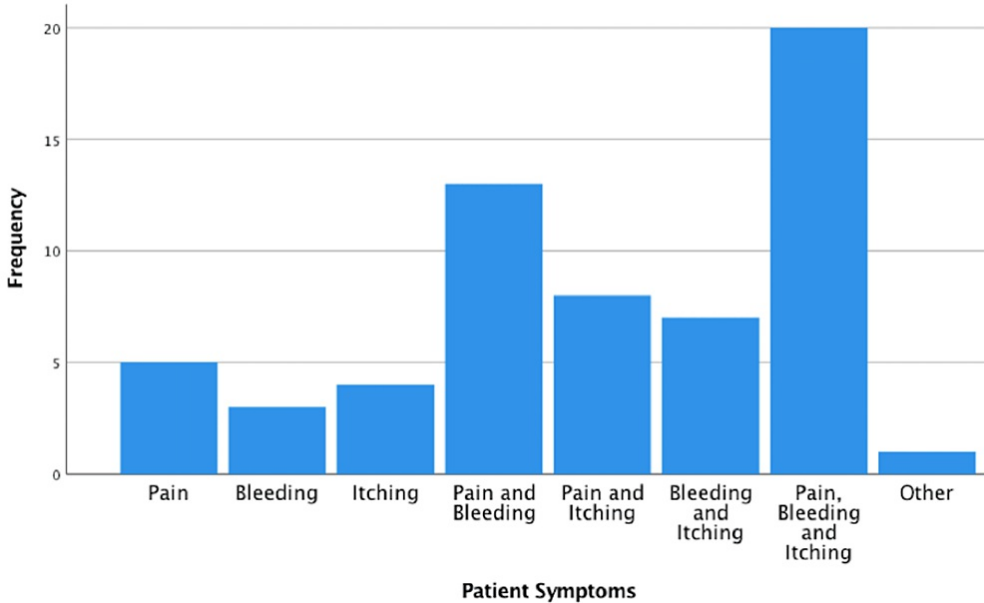
Patient symptoms and their relative frequency in the postnatal cohort

In the postnatal patient cohort, 27/156 (17%) first noticed symptoms in their first trimester, 42/156 (27%) in the second trimester, 53/156 (34%) in the third trimester up to 36 weeks, 17/156 (11%) from beyond 36 weeks and a further 17/156 (11%) first noticed haemorrhoids or anal fissures at or after delivery. One-third of the postnatal patients (52/156) reported pain, pruritis and bleeding and the rest of patients had a combination of one or more of the symptoms (see Figure 3). 73/156 (47%) patients reported symptoms improving over the pregnancy, 34/156 (22%) reported symptoms staying the same and 48/156 (31%) said they worsened over the pregnancy. 70/156 (45%) patients’ symptoms resolved within days, 42/156 (27%) in weeks and 44/156 (28%) within months. Table 3 displays the person treating the HAF in the postnatal cohort. 41/64 (64%) postnatal patients treated the condition themselves.

**Table 3:** Person treating the haemorrhoids and anal fissures (postnatal patients)

	n	Percentage
General Practitioner	15	23
General Surgeon	0	0
Patient (self-treated)	41	64
Midwife	6	9
Obstetrician	2	3

## Discussion

The most striking finding from this study is that most patients treat the condition themselves without either seeking or receiving guidance from medical professionals. This may be due to a combination of factors. Patients may find the condition embarrassing, or they may consider it a normal part of pregnancy [12]. Educating patients about HAF and options available for treatment may help change this [12]. It is also concerning that patients with bleeding, a red-flag symptom, are not seeking medical advice. The history alone is also not sufficient to distinguish haemorrhoids from anal fissures, likely leading to anal fissures being underdiagnosed.

When comparing those with symptoms versus those without, in the antenatal patients, we found only age to be a risk factor. Patients over 35 had significantly more HAF (see Table 2). In the most comprehensive study on risk factors for HAF in the literature, 280 pregnant women were examined multiple times during pregnancy and after delivery [4]. The authors took age greater than 30 as their cut-off value and did not find a difference between patients less than 30 and over 30 in terms of HAF diagnoses. This could be due to their age difference being five years younger [4]. 

In terms of the natural course of the disease, it was encouraging that 45% (70/156) of patients’ symptoms resolved within a few days. This will help when counselling patients with distressing symptoms. Our survey did not capture beyond the immediate postnatal period but, from the literature, it is important to note that some patients do experience ongoing symptoms. In one study, they found that of those women who still experienced symptoms three weeks after birth, half of these women were still having symptoms 1.5 years later [1]. In another study, it was found that 16% of women have HAF symptoms six months after delivery [2]. This survey, therefore, supports the use of preventative conservative measures to reduce the chance of long-term symptoms.

In terms of treatments, patients often self-treated (64%, 41/64), as mentioned above. 58 and 63 patients increased their fibre and fluid intake, respectively, and found these treatments effective (see Table 1). More dietary fibre has been shown to be a highly effective treatment in early haemorrhoidal disease, as recommended by the European Society for Coloproctology [10]. In a Cochrane review of seven randomised controlled trials, fibre-based laxatives were shown to be an effective and safe treatment, with haemorrhoids halved [13].

Another effective conservative treatment used by 22 patients in this study was Sitz bath salts. These have also been shown to be effective but the evidence is only moderate from the literature, with no formal randomised controlled trials published to date. In a prospective comparative study of pregnant women with haemorrhoids, 284 had three times a day Sitz bath salts baths and 211 women applied topical cream. One hundred percent of the women in the bath salts group reported healing, compared to 85% in the cream group [7]. Ice packs and manual reduction are less commonly described in the literature. The importance of toilet sitting position is highlighted in the literature [10], though, but no formal large studies have been carried out in pregnant women.

In terms of medical treatments, 38 patients tried Scheriproct topical and 17 tried Scheriproct suppositories and found them useful. Scheriproct contains steroids and a local anaesthetic. The evidence for this, as for Sitz bath salts, is limited in the literature. In a BMJ Clinical Evidence review, the advice given was that these topical treatments can be used when patients are in pain but there is no good quality evidence for their use [14]. Occasionally, contact dermatitis can occur and patients should be warned of this. In a Cochrane review of non-surgical treatment options in patients with haemorrhoids in pregnancy, oral rutoside medications were found to be effective. There is limited information on their safety profile in pregnancy, yet, though [15].

In a Swedish prospective comparative study, they assigned 496 pregnant women to a normal standard of care versus a “flexible sacrum” method of delivery where the patient was either “kneeling, standing, lying on the side, on a birth seat, or resting on all-fours”. They found fewer haemorrhoidal symptoms three weeks after birth in the intervention group [1]. This study was not randomised and subject to bias so results must be interpreted with caution but further studies in this area may reduce haemorrhoids or anal fissures associated with labour. Only one patient in this study underwent surgery for HAF during their pregnancy. This is consistent with the literature: Surgery for haemorrhoids or anal fissures is only recommended when conservative treatment options have been tried and have not worked [8,10]. If surgery is required, it should ideally be delayed until the foetus is viable [8]. The title of a BMJ editorial summarises this point well: “How to treat haemorrhoids: Prevention is best; haemorrhoidectomy needs skilled operators” [16].

Limitations

This was a survey and some of the patients had self-diagnosed HAF without a formal examination. It is possible that anal fissures are under-reported. According to the European Coloproctology Guidelines for Haemorrhoids [10], haemorrhoids should be diagnosed by history and examination. Without an examination, the diagnosis by symptoms alone can be inaccurate [17]. The limitation of this study is a limitation in the current management of HAF during pregnancy, though. With more information provided to patients, they would be more likely to inform healthcare practitioners about their symptoms and would be more likely to be formally examined and diagnosed [12]. In addition, it is worth highlighting here that flexible sigmoidoscopy without sedation, rectoscopy, anoscopy, or proctoscopy should be considered, according to European, Portuguese and Australian guideline papers on haemorrhoids [9-11]. There is some concern about the safety of these procedures during pregnancy but the complication rate in pregnancy has been shown to be very low when performed by experienced operators [18]. In addition, patients were only recruited who were physically approached in the clinic. The postnatal cohort was limited to women immediately post-delivery or re-attending with complications of delivery or recovering from these. This led to inherent selection bias in the postnatal cohort. A strength of the study was the relatively large cohort of antenatal and postnatal patients.

## Conclusions

HAF in pregnancy and postpartum can be distressing for patients and significantly affect their quality of life. This study reveals that most patients diagnose and treat the condition themselves without either seeking or receiving guidance from medical professionals. Patient education about the condition and treatment options in antenatal clinics may help change this. There is good evidence that fibre-based laxatives can reduce symptoms and a high-fibre diet and increased water intake should be encouraged.
